# Role of Nanoparticles in Enhancing Crop Tolerance to Abiotic Stress: A Comprehensive Review

**DOI:** 10.3389/fpls.2022.946717

**Published:** 2022-11-02

**Authors:** Mohamed T. El-Saadony, Ahmed M. Saad, Soliman M. Soliman, Heba M. Salem, El-Sayed M. Desoky, Ahmad O. Babalghith, Amira M. El-Tahan, Omar M. Ibrahim, Alia A. M. Ebrahim, Taia A. Abd El-Mageed, Ahmed S. Elrys, Alaa A. Elbadawi, Khaled A. El-Tarabily, Synan F. AbuQamar

**Affiliations:** ^1^Department of Agricultural Microbiology, Faculty of Agriculture, Zagazig University, Zagazig, Egypt; ^2^Department of Biochemistry, Faculty of Agriculture, Zagazig University, Zagazig, Egypt; ^3^Department of Internal Medicine and Infectious Diseases, Faculty of Veterinary Medicine, Cairo University, Giza, Egypt; ^4^Department of Poultry Diseases, Faculty of Veterinary Medicine, Cairo University, Giza, Egypt; ^5^Botany Department, Faculty of Agriculture, Zagazig University, Zagazig, Egypt; ^6^Department of Medical Genetics, College of Medicine, Umm Al-Qura University, Mecca, Saudi Arabia; ^7^Department of Plant Production, Arid Lands Cultivation Research Institute, The City of Scientific Research and Technological Applications, SRTA-City, Alexandria, Egypt; ^8^School of Life Sciences, Jiangsu Key Laboratory for Microbes and Genomics, Nanjing Normal University, Nanjing, China; ^9^Department of Soils and Water, Faculty of Agriculture, Fayoum University, Fayoum, Egypt; ^10^Department of Soil Science, Faculty of Agriculture, Zagazig University, Zagazig, Egypt; ^11^Department of Biology, College of Science, United Arab Emirates University, Al Ain, United Arab Emirates; ^12^Khalifa Center for Genetic Engineering and Biotechnology, United Arab Emirates University, Al Ain, United Arab Emirates; ^13^Harry Butler Institute, Murdoch University, Murdoch, WA, Australia

**Keywords:** abiotic stress, crop yield, modern agriculture, nanoparticles, nanotechnology, plant performance

## Abstract

Plants are subjected to a wide range of abiotic stresses, such as heat, cold, drought, salinity, flooding, and heavy metals. Generally, abiotic stresses have adverse impacts on plant growth and development which affects agricultural productivity, causing food security problems, and resulting in economic losses. To reduce the negative effects of environmental stress on crop plants, novel technologies, such as nanotechnology, have emerged. Implementing nanotechnology in modern agriculture can also help improve the efficiency of water usage, prevent plant diseases, ensure food security, reduce environmental pollution, and enhance sustainability. In this regard, nanoparticles (NPs) can help combat nutrient deficiencies, promote stress tolerance, and improve the yield and quality of crops. This can be achieved by stimulating the activity of certain enzymes, increasing the contents (e.g., chlorophyll) and efficiency of photosynthesis, and controlling plant pathogens. The use of nanoscale agrochemicals, including nanopesticides, nanoherbicides, and nanofertilizers, has recently acquired increasing interest as potential plant-enhancing technologies. This review acknowledges the positive impacts of NPs in sustainable agriculture, and highlights their adverse effects on the environment, health, and food chain. Here, the role and scope of NPs as a practical tool to enhance yield and mitigate the detrimental effects of abiotic stresses in crops are described. The future perspective of nanoparticles in agriculture has also been discussed.

## Introduction

Abiotic stress factors, that can affect modern agricultural productivity worldwide include high or low temperature, waterlogging, drought, salinity, heavy metals (HMs), and ultraviolet (UV) radiation (Seleiman et al., 2020a; Badawy et al., 2021). Plant response to abiotic stress involves alterations in various morphological, physiological, and biochemical processes depending on the crop type, stress type, and time of exposure (Semida et al., 2014; Desoky et al., 2020a; Rady et al., 2021; Abd El-Mageed et al., 2022; Shaaban et al., 2022). As such, sustainable agriculture and yield productivity can improve the quality of soil, water, and other resources required by plants (Badal et al., 2013; Saxena et al., 2016; Desoky et al., 2020b). To meet the increasing global food demand, researchers are striving to ameliorate the detrimental effects of abiotic stresses, enhance crop yield and food production, and achieve sustainability and food security. Indeed, for addressing these urgent global concerns, researchers must continue developing innovative technologies or solutions.

Nanotechnology is a fascinating and rapidly developing branch of research that has led to various innovations (El-Saadony et al., 2020, 2021a; Abd El-Ghany et al., 2021). In particular, nanotechnology can help provide effective solutions to agriculture-related problems and achieve a sustainable and secure future for agriculture (Seleiman et al., 2021b). Nanotechnology has gained tremendous attention in recent years owing to its wide range of applications in medicine, drug delivery, energy, poultry production, and the agrifood sector (Seleiman et al., 2020a; Yousry et al., 2020; Salem et al., 2021). In agriculture, nanotechnology is mainly utilized in the application of nanofertilizers and nanopesticides to track products and nutrient levels for enhancing growth and productivity and increasing plant resistance to insect pests and microbial diseases (Shang et al., 2019; Bhatt et al., 2020).

Nanoparticles (NPs) are tiny materials 1–100 nm in size (Khan and Upadhyaya, 2019). In contrast to their larger sized equivalents, NPs possess certain unique and diverse physicochemical properties. For instance, NPs have a large surface area-to-volume ratio, high adsorption efficacy, and increased connecting and working efficiencies owing to their extremely small size (Nel et al., 2006; Dubchak et al., 2010). Thus, NPs have been integrated into disease management strategies as bactericides/fungicides/pesticides to enhance plant health. NPs can also serve as macro and micro-nanofertilizers in plants to alleviate nutrient deficiency symptoms and supplement essential elements. Various biological, physical, and chemical techniques can be used for NPs synthesis (Singh et al., 2016).

**FIGURE 1:**
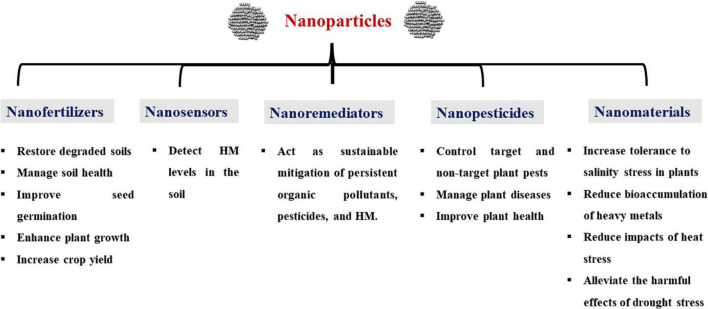
Potential applications of nanotechnology in agriculture. HM, heavy metals.

In agriculture and agrifood business, NPs can be applied in the form of nanosensors, nanofertilizers, nanoherbicides/nanopesticides, and nanoremediators (Figure 1; Elsakhawy et al., 2018; El-Saadony et al., 2021b). However, the mechanisms of underlying how NPs interact with plants have not been completely elucidated (Saxena et al., 2016; Khan et al., 2019). Therefore, this review highlights the current knowledge and potential uses of NPs that are widely used in agriculture, along with their effects on plants for better crop improvement and sustainable agriculture.

## Abiotic Stress

**FIGURE 2:**
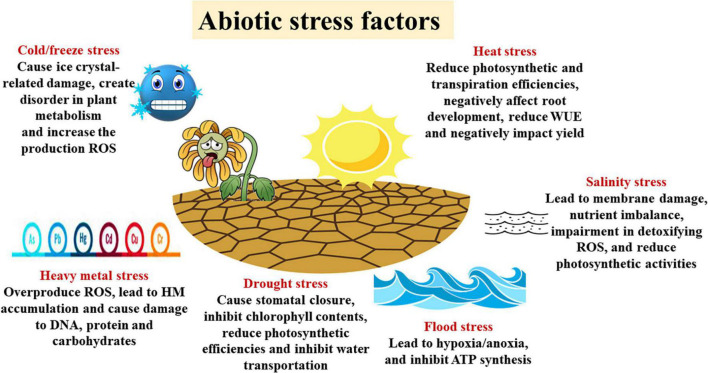
Abiotic stress factors and their negative effects on plants. ROS, reactive oxygen species; WUE, water use efficiency; HM, heavy metals.

With the increasing world population, abiotic stress conditions are increasingly affecting crop production (Figure 2). During stress, physiological and biochemical changes occur in plant cells that can adversely affect plant growth, development, and productivity (Al-Ashkar et al., 2019; Seleiman et al., 2020b,2021a; Taha et al., 2021). Species and varieties bred to tolerate these challenges along with nanotechnology and other climate-sensitive agricultural technologies could be the most efficient adaptation strategy to cope with climate and abiotic stress factors, thereby achieving sustainable production (Kumari et al., 2022).

Major abiotic stressors that limit crop returns globally include heat stress and drought (Abd El-Mageed et al., 2019; Batool et al., 2020; Semida et al., 2020; Rasheed et al., 2021). Drought induces many morphological, physiological, molecular, and metabolic changes in plants which are relatively significant. Plants regulate their stomatal conductance to control the amount of water lost, and optimize CO_2_ assimilation to avoid photosynthetic inhibition, allowing them to resist water stress (Faraji and Sepehri, 2020). Under arid conditions, phenolics, flavonoids and antioxidant enzymes are affected to a large extent. Root-sourced signals are transported *via* the xylem to leaves, thus, affecting the cellular status in drought-stressed plants (Afshari et al., 2021). Turgor loss can also be observed in plants under drought stress. If dehydration is severe, the protoplasm may become rigid, consequently altering the cellular metabolism and inhibiting plant growth. Drought can severely disrupt cellular metabolism, ion accumulation, membrane structure integrity, and protein structures in plants. Therefore, leaf growth, photosynthetic rates and enzymatic activities are reduced. Drought can also induce the excessive generation of reactive oxygen species (ROS) in plants; and thereby results in oxidative stress (Cruz de Carvalho, 2008). In addition, salinity and HMs stress are also considered among the environmental factors that limit crop yield in many countries (Abd El-Mageed et al., 2018; Ye et al., 2019; Sofy et al., 2020; Taha et al., 2020; Dustgeer et al., 2021; Khan et al., 2021; Seleiman et al., 2022).

Salinity is a type of abiotic stress that is widespread and responsible for considerably decreasing plant growth. Soil salinity inhibits seed germination owing to the low osmotic potential generated around the seeds, which prevents water uptake (Tavakkoli et al., 2010; Seleiman et al., 2020c; Alkharabsheh et al., 2021; Taha et al., 2021). Sodium chloride (NaCl)-induced oxidative stress in legumes, considerably inhibits growth, decreases seed nutrient quality and lowers nodulation (Hernandez et al., 2000; Ahmad et al., 2008). Plants can employ various antioxidant defense mechanisms, both enzymatic and non-enzymatic, to reduce the effect of oxidative stress associated with salinity. Ascorbate and carotenoids are critical non-enzymatic defense mechanisms against salinity, whereas proline (Pro) is a known osmoregulatory stress-related compound (Anoop and Gupta, 2003).

Plant growth and development benefit from essential elements, such as cobalt (Co), copper (Cu), iron (Fe), manganese (Mn), molybdenum (Mo), nickel (Ni), zinc (Zn), and non-essential elements such as cadmium (Cd), chromium (Cr), lead (Pb), and mercury However, all HMs are highly toxic to plants at high concentrations (White and Pongrac, 2017). The toxic levels of HMs adversely affect various metabolic processes. This may include, but not be limited to, degradation or displacement of protein structures resulting from the development of bonds between the HMs and sulfhydryl groups (Hall, 2002); disruption of cytoplasmic membrane integrity (Farid et al., 2013), and suppression of photosynthesis, respiration, and enzymatic actions (Hossain et al., 2012).

## NPs

**FIGURE 3:**
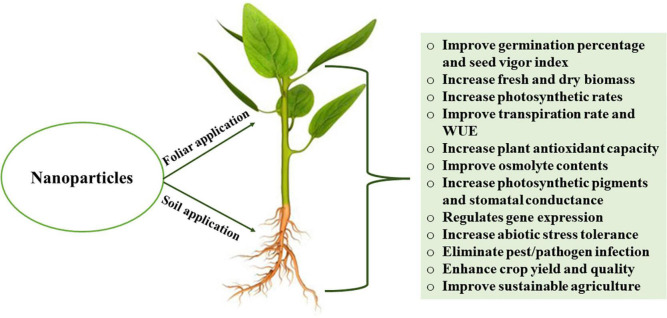
Effects of nanoparticles on plant health, growth performance and physiological parameters. WUE, water use efficiency.

NPs are microscopic particles that can enter the cell through aboveground plant organs (cuticle, epidermis, stomata, hydathodes, or other openings) or underground organs (root tips, cortex, lateral root, wounds, or other openings). The physiological and morphological effects of NPs vary according to plant species, development period, development agents, application method, dose, and exposure time (Dietz and Herth, 2011; Rizwan et al., 2017). According to the mass flow/pressure flow hypothesis, NPs that enter through the stomata are carried within the plant by the phloem and are transported *via* pressure differences between the leaves and roots (Turgeon, 2010). The route through which NPs enter the plants alters many plant processes, including germination, antioxidant activity, macro and micronutrients, chlorophyll content, chloroplast number, and photosynthesis (Cinisli et al., 2019). In *Arabidopsis*, NPs application altered intraroot signals by affecting ethylene production (Syu et al., 2014). NPs can penetrate the cell membrane and the cell wall in order to be transported to the epidermis, xylem, central cylinder, and leaves (Tripathi et al., 2017a). Before reaching the central cylinder, NPs are passively transported in the endodermis (Judy et al., 2012). NPs are transported *via* the active route through osmotic pressure, capillary forces, cell wall pores, and plasmodesmata in plant roots or *via* the symplastic route (Usman et al., 2020). In general, NPs can bind to carrier proteins *via* ion channels, aquaporin, and endocytosis, as well as disrupt the plasma membrane to induce the formation of pores for crossing into the cells. The passage of NPs through the cell wall relies on their pore size. Small-sized NPs simply pass through the cell wall (Fleischer et al., 1999), while larger NPs pass through the hydathodes, stigma, and stomata (Hossain et al., 2016). NPs are transported *via* the stomata when their dimensions are < 15–40 nm (Eichert et al., 2008). Such NPs can act as a substitute for the vascular cambium, in the stomata and be transferred to various plant compartments through the phloem (Tripathi et al., 2017a). The NPs that are widely used in agriculture and their role in enhancing crop tolerance to abiotic stress (Figure 3) are summarized in Table 1. In seed coating, NPs enter through parenchymatous intercellular spaces in the seed coat in which aquaporins play an important role in controlling NPs entry (Abu-Hamdah et al., 2004; Lee et al., 2010).

**TABLE 1:** Most commonly used nanoparticles in agriculture and their impacts in enhancing crop tolerance to abiotic stress.

Nanoparticles	Administration	Plants species	Abiotic stresses	Impact	References
Zn	Nano-zinc solution was added to petri dishes containing soybean seeds	Soybean	Drought	Increased the germination rate and reduced seed residual weight	Sedghi et al., 2013
	Added to pot soil and the soil was mixed to uniform nanoparticles	Barley	Drought	Stimulated growth, improved production yield, and fortified edible grains with crucial nutrients and increased N acquisition	Dimkpa et al., 2019
	Nano-ZnO suspension were added into half of these pots at 7 days after emergence	*Zea mays* L. cv. Jidan 27	Drought	Enhanced melatonin synthesis. Promoted the functioning of the antioxidant system	Sun et al., 2020
	Foliar application to leaf area	Sunflower and soybean	Salinity	Reduced Na levels in leaves and increased Zn levels, substomatal CO_2_ concentration, the CO_2_ acclimatization ratio, chlorophyll content, and Fv/Fm	Torabian et al., 2016
	Nano-ZnO suspension were added into half of these pots	*Lupinus termis*	Salinity	Initiated plant growth; restored the levels of photosynthetic pigments, organic solutes, total phenols, ascorbic acid, and antioxidant enzymes; and decreased MDA level	Abdel Latef et al., 2017
	Foliar application to leaf area	Wheat	Salinity	Improved plant development	Fathi et al., 2017
	Foliar application to leaf area	Tomato	Salinity	Enhanced antioxidant enzyme levels. Promoted root and shoot growth. Increased biomass and photosynthetic pigment contents	Faizan et al., 2021
	Foliar application to leaf area	*Trigonella foenum-graecum*	Salinity	Enhanced protein and Pro contents, promoted antioxidant activity, and reduced H_2_O_2_ and MDA levels	Noohpisheh et al., 2021
	Foliar application to leaf area	*Mangifera indica* L.	Salinity	Increased the contents of antioxidant enzymes, total sugars, and Pro	Elsheery et al., 2020
	Foliar application to leaf area	Pearl millet	Mineral nutrient	Increased shoot and root length, root area, chlorophyll content, total soluble leaf protein, plant dry biomass, enzymatic activity, growth, and net photosynthesis	Tarafdar et al., 2014
	Foliar application	*Coffea arabica*	Mineral nutrient	Increased growth, biomass, and net photosynthesis	Rossi et al., 2019
	Nano-ZnO suspension were added into pots	Wheat	Mineral nutrient	Improved grain production and biomass	Du et al., 2019
	ZnO particles were sprayed by foliar (25-mL per pot) after 2 weeks of germination	*Cyamopsis Tetragonoloba*	Mineral nutrient	Stimulated plant development; increased biomass, nutrient and chlorophyll contents, levels of soluble protein, phytase, phosphatase, and alkaline phosphatase; and enhanced enzymatic activity	Raliya and Tarafdar, 2013
	Added to seedlings’ nutrient solution	*Nicotiana tabacum*	Mineral nutrient	Positively impacted growth physiology and increased metabolite levels, enzymatic activities, and the anatomical features of plants	Tirani et al., 2019
	The soil was amended with the NPs suspensions (well mixed) and kept 24 h for stabilization.	Pea	Mineral nutrient	Increased root length	Mukherjee et al., 2014
	The seeds were soaked in different Zn NPs suspension	Rice	Mineral nutrient	Enhanced germination and antioxidant activity in plants	Panda, 2017
	Added to seedling nutrient solution	*Arabidopsis thaliana*	Mineral nutrient	Modulated the transcription of different genes involved in Zn uptake, macronutrient and micronutrient homeostasis, and hormone control	Nair and Chung, 2017
	Foliar application	*Brassica juncea*	Mineral nutrient	Increased plant development and improved antioxidant levels	Nayan et al., 2016
	The seeds were soaked in different Zn NPs suspension	*Arachis hypogaea*	Mineral nutrient	Increased seed germination and seedling vigor	Prasad et al., 2012
	The seeds were primed in different Zn NPs suspension	*Lupinus termis*	Mineral nutrient	Modulated growth, photosynthesis, and antioxidant responses	Abdel Latef et al., 2017
	Foliar application	Eggplant	Mineral nutrient	Increased RWC and photosynthetic pigments. Enhanced fruit yield and growth parameters	Semida et al., 2021
	Added to seedling nutrient solution	*Oryza sativa* L.	HMs	Increased phytochelatin content and promoted growth	Yan et al., 2021
	Sprayed to plant foliage every alternate day for 2 weeks	Soy bean		Increased photosynthetic pigments, and induced antioxidant enzymes. Enhanced growth	Ahmad et al., 2020
	Foliar application	Maize		Increased biomass, photosynthetic pigments, and antioxidant enzymes	Rizwan et al., 2019a
	Foliar application	Wheat		Increased Cd uptake and induced antioxidant enzymes. Enhanced growth	Rizwan et al., 2019b
	Added to seedling nutrient solution	*Lycopersicon leucocephala*		Stimulated the antioxidant system. Mitigated lipid peroxidation. Enhanced protein and pigment contents. Enhanced growth	Venkatachalam et al., 2017
	Foliar application	Common bean	Salinity	Enhanced antioxidant enzyme activity and mitigated salinity-induced adverse effects	El-Saadony et al., 2021c
Si	Plants were irrigated with silica nanoparticles for 2 weeks	*Ocimum basilicum*		Enhanced pigment and Pro levels. Improved growth parameters	Kalteh et al., 2014
	Added to nutrient solution	*Lens culinaris* Medik.		Inhibited seed activity and germination and reduced FW	Sabaghnia and Janmohammadi, 2014
	Added to petri dishes containing seeds	*Cucurbita pepo* L.		Increased seed germination. Reduced H_2_O_2_ and MDA levels. Improved electrolyte levels. Increased photosynthetic pigments and antioxidant enzymes. Enhanced growth	Siddiqui et al., 2014
	Soaking seed in silicon nanoparticles suspension	*Solanum lycopersicum* L.		Increased the seed germination rate. Increased photosynthetic pigments. Regulated salt toxicity-associated genes. Improved root growth and root weight. Upregulated the expression of four salt stress genes (*AREB*, *TAS14*, *NCED3*, and *CRK1*) and downregulated that of six salt stress genes (*RBOH1*, *APX2*, *MAPK2*, *ERF5*, *MAPK3*, and *DDF2*). Inhibited salinity-induced adverse effects on the seed germination rate and seed development	Haghighi and Pessarakli, 2013; Almutairi, 2016a
	Added to petri dishes containing seeds	*Lycopersicum esculentum*		Increased seed germination. Improved root growth and root weight	Haghighi et al., 2012
	Foliar application	*Vicia faba* L.		Stimulated the antioxidant system. Increased crop yield and RWC	Qados, 2015
	In irrigation water and foliar application	*Fragaria* sp.		Increased chlorophyll and Pro contents. Increased RWC	Avestan et al., 2019
	Added to nutrient solution of seedling	Hawthorn	Drought	Improved plant tolerance by retaining critical physiological and biochemical functions. Exhibited non-significant effects on chlorophyll and carotenoid contents	Ashkavand et al., 2015
	Foliar application	Higher plants	Salinity, drought	Enhanced antioxidant enzyme activity and promoted plant stress resistance	Liang et al., 2007
	Added to nutrient solution of seedling	*Crataegus* sp.		Increased biomass and photosynthetic pigment levels. Enhanced net photosynthesis and stomatal conductance by upregulating photosynthesis	Ashkavand et al., 2015
	Added to nutrient solution of shoots	*Musa acuminata*		Maintained Na^+^–K^+^ balance. Promoted photosynthesis. Increased chlorophyll levels and leaf growth	Mahmoud et al., 2020
	Foliar application	Tomato	Salinity	Increased germination, root length, DW, chlorophyll content, Pro precipitation, the photosynthetic rate, and leaf water content. Regulated antioxidant enzyme activity	Haghighi et al., 2012; Haghighi and Pessarakli, 2013; Haghighi and Pourkhaloee, 2013
	Foliar application	Basil	Salinity	Increased FW and DW and chlorophyll and Pro contents. Increased antioxidant enzyme activity	Kalteh et al., 2014; Siddiqui et al., 2014
	Added to nutrient solution	Lentil	Salinity	Increased seed germination and seedling development. Improved various defense mechanisms	Sabaghnia and Janmohammadi, 2014
	Added to petri dishes containing seeds	Squash	Salinity	Increased germination and growth indices and enhanced the overall defense mechanism and antioxidant enzyme activity. Enhanced photosynthetic parameters	Siddiqui et al., 2014
	Foliar application	Broad bean	Salinity	Increased seed germination and development, antioxidant enzyme activity, RWC and total production yield, total soluble sugars, and membrane strength	Qados, 2015; Qados and Moftah, 2015
	Added to the nutrient solution immediately after the plants were transplanted.	Maize	Salinity	Increased FW	Gao et al., 2006
	Added in irrigation water	*Prunus mahaleb*	Salinity	Increased seed germination and development, photosynthetic parameters, and antioxidant enzyme activity. Reduced MDA and H_2_O_2_ levels, chlorophyll destruction, and oxidative destruction	Ashkavand et al., 2018
	Added in irrigation water	Sweet pepper	Salinity	Markedly regulated plant tolerance	Tantawy et al., 2015
	Foliar application	Peregrina	Salinity	Increased growth and chemical constituents. Reduced Na and Cl precipitation and total phenolic and flavonoid content in leaves	Ashour and Abdel Wahab, 2017
	Plants were irrigated with silica nanoparticles for 2 weeks	Basil	Salinity	Increased biomass and chlorophyll and Pro contents	Kalteh et al., 2014
	Applied In irrigation water	Cucumber	Salinity	Elevated plant germination and growth parameters. Improved nutrient absorption and fruit production	Alsaeedi et al., 2018, 2019
	Applied In irrigation water	Cucumber	Water deficit, salinity	Improved growth and productivity by alerting the plant of the nutrient uptake, such as increased N and K levels	Alsaeedi et al., 2019
	Foliar application	Soybean	Salinity	Decreased oxidative damage through the expression of antioxidative enzymes	Farhangi-Abriz and Torabian, 2018
	Applied In irrigation water	Soybean	Hg toxicity	Immobilized and inactivated Hg	Li et al., 2020
	Added to petri dishes containing seeds	Common bean	Na^+^ stress	Improved the germination percentage, vigor index, seed germination rate, and the length and dry mass of shoots and roots.	Alsaeedi et al., 2017
	Foliar application	Rice	HMs	Reduced HMs toxicity and promoted development by reducing bioaccumulation and translocation of HMs in plants	Wang et al., 2016
	Applied In irrigation water	Pea	Cr (VI)	Decreased Cr (VI) absorption, promoted antioxidant defense mechanisms, and increased nutrient precipitation	Delfani et al., 2014
	Added to nutrient solution of seedling	Pea	Cr (VI)	Alleviated Cr-induced phytotoxicity and improved overall growth potential	Tripathi et al., 2015b
	Seed soaking	Maize	As	Mitigated As toxicity and exhibited increased As resistance in maize cultivar compared to maize hybrid	Tripathi et al., 2016
	Foliar application	Safflower	Mineral nutrient	Enhanced production yield	Janmohammadi et al., 2016
	Added to petri dishes containing seeds	Wheatgrass	Cold	Averted seed dormancy and improved seed germination and seedling weight	Azimi et al., 2014
	Foliar application	Wheat	UV-B radiation	Alleviated UV-B radiation stress in seedlings and resulted in improved protection of wheat seedlings *via* NO-mediated antioxidant defense, consequently counterbalancing toxic ROS generation	Tripathi et al., 2017b
	Foliar application	Wheat	Cd toxicity	Increased SOD and POD activity	Ali et al., 2019
	Seed priming	Wheat	Cd toxicity	Mitigated oxidative stress, positively affected antioxidant enzyme activity, decreased Cd concentration mainly in grains, and increased Si concentration in plants	Hussain et al., 2019a,b
	Added to soil	Maize	UV-B radiation	Enhanced the growth and physiological responses	Suriyaprabha et al., 2012
Ag	Silver nanoparticles (15 ml) per every test plantlets was carried out for 14 days	Lentil	Drought	Decreased the germination ratio, root length, FW, and DW	Hojjat and Ganjali, 2016
	Seed soaking	Tomato	Salinity	Increased the germination percentage and germination ratio, root length, and seedling FW and DW. Upregulated four genes (*AREB*, *MAPK2*, *P5CS*, and *CRK1*) and downregulated three genes (*TAS14*, *DDF2*, and *ZFHD1*), thereby alleviating salt stress.	Almutairi, 2016b
	Seed soaking	*Satureja hortensis* L.		Increased germination percentage and enhanced growth parameters, such as shoot length. Improved salt stress tolerance	Nejatzadeh, 2021
	Seed priming	Wheat	Salinity	Mitigated the damaging impacts of salinity stress	Mohamed et al., 2017; Abou-Zeid and Ismail, 2018
	Foliar application			Enhanced seed germination efficiency. Mitigated oxidative stress. Induced antioxidant enzymes	Wahid et al., 2020
	Seed priming			Increased plant growth hormones, including NAA, IBA and ABA. Promoted growth	Abou-Zeid and Ismail, 2018
	Silver nanoparticles (15 ml) per every test plantlets was carried out for 14 days			Promoted seed germination efficiency. Increased FW and DW	Hojjat and Kamyab, 2017
	Foliar application			Mitigated salt-stress–induced oxidative damage by inducing antioxidant enzymes. Regulated salt tolerance	Wahid et al., 2020
	Seed priming			Increased total sugar and Pro contents	Mohamed et al., 2017
	In irrigation water		Heat	Increased the leaf number and promoted growth	Iqbal et al., 2019
	Applied In irrigation water	Bok choy	Cd	Improved biomass, chlorophyll content, and vitamin C levels as well as SOD, CAT, and POD activity and reduced MDA levels	Li and Huang, 2014
	Added in nutrient solution of seedling	*Arabidopsis thaliana*	Cold	Activated and increased the expression of antioxidant genes (*Me*Cu/ZnSOD and *Me*APX2)	Kohan-Baghkheirati and Geisler-Lee, 2015
	Added in nutrient solution of seedling	*Phaseolus vulgaris* L.		Enhanced seedling quality and increased net photosynthesis. Regulated cold stress tolerance	Prazak et al., 2020
	In irrigation water	Wheat	Heat	Improved plant development and heat resistance	Iqbal et al., 2019
	Spray	Horse-shoe pelargonium	Dark	Increased antioxidant enzyme activities, photosynthetic pigment content, and petal longevity. Reduced lipid peroxidation and petal abscission	Hatami and Ghorbanpour, 2013, 2014
	Added in nutrient solution of seedling	Soybean	Flooding	Decreased the formation of cytotoxic byproducts of glycolysis as well as increased the abundance of stress-related proteins and seedling growth	Mustafa et al., 2015
	Seed soaking	Saffron	Flooding	Blocked ethylene signaling, promoted root growth, and increased leaf DW and root length	Rezvani et al., 2012
	Spraying	*Chrysanthemum*	Post-harvest	Enhanced the vitality and succulence of cut flowers and reduced FW loss and stem bacterial count	Kazemipour et al., 2013
	In nutrient media	*Brassica juncea*	–	Improved growth and antioxidant potential *in vitro*	Sharma et al., 2012a
	In nutrient solution	Rice	–	Improved photosynthetic pigment content and enhanced CAT, APX, and GR activity	Gupta et al., 2018
	In nutrient media	*Arabidopsis thaliana*	–	Improved anthocyanin precipitation in seedlings and stimulated protein precipitation	Syu et al., 2014
	Spraying	Fenugreek	–	Increased shoot length, leaf number, plant number, and the production of photosynthetic pigments, phenolics, flavonoids, and tannins	Sadak, 2019
	Foliar application	Cucumber	–	Activated antioxidant processes, enhanced phenolic features, and altered membrane characteristics	Zhang et al., 2018
	Foliar application	*Vigna sinensis*	–	Promoted growth and increased biomass by increasing root nodulation and soil bacterial diversity	Pallavi et al., 2016
TiO_2_	Foliar application	Wheat	Drought	Improved development, productivity, seed gluten, and starch content. Increased growth and starch content. Increased seedling DW. Increased chlorophyll and carotenoid contents, RWC, the transpiration rate, and stomatal conductance	Jaberzadeh et al., 2013; Faraji and Sepehri, 2020
	In irrigation water	*Linum usitatissimum*		Reduced H accumulation and increased chlorophyll and carotenoid contents and 2O_2_ and MDA levels	Aghdam et al., 2016
	In irrigation water	*Ocimum basilicum* L.		Mitigated drought-induced adverse effects and increased biomass and RWC	Kiapour et al., 2015
	Exogenous application	*Vigna radiata* L.	HMs	Induced antioxidant activity. Reduced MDA levels. Improved growth and increased biomass	Katiyar et al., 2020
	Added in the soil	*Glycine max* L.		Mitigated Cd toxicity. Reduced lipid peroxidation. Increased chlorophyll content and reduce Pro content. Increased RWC, growth parameters, and net photosynthesis	Singh et al., 2016
	In irrigation water	Linseed or flax	Drought	Increased photosynthetic pigment content, plant development, production yield and reduced H_2_O_2_ and MDA levels	Aghdam et al., 2016
	In irrigation water	Basil	Drought	Decreased the negative effects of drought stress	Kiapour et al., 2015
	Spraying on shoot	Moldavian dragonhead	Drought (oxidative stress)	Alleviated oxidative stress. Increased Pro precipitation, and reduced H_2_O_2_ and MDA levels.	Mohammadi et al., 2016
	Foliar application	Broad bean	Salinity	Increased plant development by improving antioxidant enzyme activities and increasing the levels of soluble sugars, amino acids, and Pro and other metabolites, thereby contributing to osmoprotection.	Abdel Latef et al., 2018
	Seed priming	*Zea mays* L.		Enhanced seed germination efficiency Decreased Na^+^, Pro, and MDA levels and increased K^+^. Increased phenolic and antioxidant contents and RWC. Increased FW and DW.	Shah et al., 2021
	Added three times (three continuous days) to Hoagland solution 2 weeks after salinity stress application.	*Dracocephalum moldavica*		Positively impacted physiochemical properties by inducing antioxidant activities.	Gohari et al., 2020
	In nutrient solution	Chickpea	Cold	Improved antioxidative enzyme activities and reduced H_2_O_2_ levels and electrolyte leakage TiO_2_ precipitation was increased in the cold-sensitive genotype compared to the cold-tolerant genotype.	Mohammadi et al., 2013, 2014
	TiO2 suspension was added to Petri dish containing seeds		Cold	Increased the expression of genes encoding Rubisco- and chlorophyll-binding proteins, decreased H_2_O_2_ levels, and increased the activity of phosphoenolpyruvate carboxylase	Hasanpour et al., 2015
	Soil and irrigation		Cold	Altered metabolic pathways as observed through transcription profiling	Amini et al., 2017
	Spraying	Tomato	Heat	Increased photosynthesis by regulating energy dissipation and caused leaf cooling by increasing stomatal opening	Qi et al., 2013
	In irrigation water	Flax	Drought	Improved chlorophyll and carotenoid content, improved flax growth and yield attributes, and reduced H_2_O_2_ and MDA levels	Aghdam et al., 2016
	Foliar application	Spinach	UV-B radiation	Reduced ROS and MDA levels and improved antioxidative enzyme activity and the oxygen evolution rate	Lei et al., 2007, 2008
	Seed soaking	Spinach	Excessive light	Increased antioxidative enzyme activity, reduced ROS and MDA levels, and improved membrane stability and maintained an intact chloroplast structure	Hong et al., 2005
	In nutrient media	*Lemna minor*	–	Increased the activities of different enzymes and eliminated accumulated ROS in plant cells	Song et al., 2012
	In irrigation water	Wheat	–	Promoted leaf health and growth kinetic traits	Dawood et al., 2019
	Spraying with hand automizer	*Salvia ocinalis*	–	Improved antioxidant action and increased phenol and flavonoid contents	Ghorbanpour, 2015
	Spraying with hand automizer	*Hyoscyamus niger*	–	Increased SOD activity and exhibited the highest alkaloid (hyoscyamine and scopolamine) content	Ghorbanpour et al., 2015
	Spraying	Wheat	–	Upregulated monosaccharides and azelaic acid, triggering tyrosine metabolism in roots. Upregulated reserve sugars and tocopherol, phenylalanine, and tryptophan pathways.	Silva et al., 2020
	Foliar application	Cotton	Drought	Increased total phenolics, total soluble proteins, total free amino acids, Pro content, total antioxidant capacity, CAT, POD, and SOD activity	Shallan et al., 2016
	In nutrient solution	Licorice	Cold	Decreased lipid peroxidation and H_2_O_2_ levels. Increased phenolics, total protein, and osmolyte contents	Kardavan Ghabel and Karamian, 2020
	Foliar application	Rice	–	Increased biomass, decreased the photosynthetic ratio, and reduced energy consumption in metabolism	Zhang et al., 2020
	Foliar application	Radish	–	Improved photosynthesis and total phenol levels	Tighe-Neira et al., 2020
CeO_2_	Spraying	Mouse-ear Cress	Salinity	Improved leaf mesophyll K^+^ retention, chlorophyll content, biomass, and photosynthesis	Wu et al., 2018
	In the soil	Canola	Salinity	Shortened root apoplastic barriers, thereby allowing increased Na^+^ transport to shoots and reduced Na^+^ accumulation. Increased plant biomass and photosynthetic apparatus efficiency	Rossi et al., 2016
Chitosan	In soil and foliar application	Wheat	Drought	Increased leaf area, RWC, chlorophyll content, photosynthetic rate, CAT and SOD activities, crop yield, and biomass	Behboudi et al., 2019
	In soil and foliar application	Barley	Drought	Increased RWC, grain weight, grain protein, Pro content, and CAT and SOD activities	Behboudi et al., 2018
Al_2_O_3_	Seed soaking	Soybean	Flooding	Regulated the ascorbate–glutathione pathway, membrane permeability, and tricarboxylic acid cycle activity	Mustafa and Komatsu, 2016
Mn	Foliar application	Bell peppers	Salinity	Improved seed germination and root growth. Altered gene expression	Ye et al., 2020
Mn_3_O_4_	Foliar application	Cucumber	Salinity	Increased photosynthetic pigment content, net photosynthesis, in biomass which resulted in alterations in metabolomes.	Lu et al., 2020
Fe	In nutrient media	Grape	Salinity	Increased total protein content and decreased Pro content, antioxidant enzymatic activity, and H_2_O_2_ levels. Decreased Na^+^ and increased K^+^	Mozafari et al., 2018a
	Foliar application	Moldavian balm	Salinity	Affected amino acid concentration and PPO, PAL, and SOD activities. Increased the gene expression of TAT, RAS, and RA	Moradbeygi et al., 2020a
	Foliar application	Moldavian balm	Salinity	Increased shoot and root leaf area, leaf length, FW, and DW	Moradbeygi et al., 2020b
	Seed soaking	Sorghum	Salinity	Improved the photosynthetic rate, chlorophyll index, PSII efficiency, RWC, and lipid peroxidation	Maswada et al., 2018
	In nutrient media	*Dracocephalum moldavica* L.		Increased phenolic compound contents and enhanced APX, GR, CAT, and GPX activities	
	In nutrient media	Fragaria × ananassa Duch.		Increased photosynthetic pigments and total sugars, Fe levels, transpiration rate, and RWC. Enhanced membrane stability. Enhanced plant growth and weight. Decreased Na^+^ levels	Mozafari et al., 2018b
	Foliar application	*Mentha piperita* L.		Decreased MDA and Pro contents. Decreased antioxidant enzymes	Askary et al., 2017
	Into soil	Wheat	Salinity and Cd	Increased photosynthetic pigments, NPK, and antioxidant enzymes activity. Increased growth, plant weight, and biomass.	Manzoor et al., 2021
	Into soil and foliar application	*Triticum aestivum* L.	Drought and Cd	Enhanced Fe uptake, and improved growth parameters and photosynthetic activities	Adrees et al., 2020
	Into soil and foliar application	*Oryza sativa* L.	Drought and Cd	Enhanced nutrient uptake and photosynthetic parameters. Enhanced growth and increased biomass	Ahmed et al., 2021
	Seed soaking	*Brassica juncea*	Cr	Controlled the conversion and accumulation of Cr (VI)	Madhavi et al., 2013
	In nutrient media	*Arabidopsis thaliana*	Drought	Promoted H^+^-ATPase activity, maintained stomatal opening and closure; increased biomass, photosynthetic pigments, and internal CO_2_	Kim et al., 2015
	Seed soaking	*Brassica napus*		Enhanced growth and increased chlorophyll levels and reduced H_2_O_2_ and MDA levels	Palmqvist et al., 2017
	Into soil and foliar application	*Triticum aestivum* L.	HMs	Reduced HMs-induced toxic effects and enhanced SOD and POX activities	Konate et al., 2017
Se	In soil and Foliar application	Wheat	Drought Heat Fungal infection	Maintained leaf water status and chlorophyll and carotenoid contents, which enhanced plant growth and increased biomass	El-Saadony et al., 2021d

*ABA, abscisic acid; Ag, silver; Al_2_O_3_, aluminum oxide; APX, ascorbate peroxidase; As, arsenic; CAT, catalase; Cd, cadmium; CeO_2_, cerium oxide; Cl, chlorine; CO_2_, carbon dioxide; Cr, chromium; DW, dry weight; Fe, iron; FW, fresh weight; GPX, glutathione peroxidase; GR, glutathione reductase; H, hydrogen; H_2_O_2_; hydrogen peroxide; Hg, mercury; HMs, heavy metals; IBA, indole-3-butyric acid; K, potassium; MDA, malondialdehyde; Mn, manganese; Mn_3_O_4_, manganese oxide; N, nitrogen; Na, sodium; NAA, 1-naphthaleneacetic acid; NO, nitric oxide; NPK, nitrogen:phosphorus:potassium; NPs, nanoparticles; PAL, phenylalanine ammonia-lyase; POD, peroxidase; POX, guaiacol peroxidase; PPO, polyphenol oxidase; PSII, photosystem II; RA, rosmarinic acid; RAS, rosmarinic acid synthase; ROS, reactive oxygen species; Pro, proline; RWC, relative water content; Se, selenium; Si, silicon; SOD, superoxide dismutase; TAT, tyrosine aminotransferase; TiO_2_, titanium dioxide; UV, ultraviolet; Zn, zinc.*

With the emergence of new nanotechnological applications, the use of nanomaterials with a high surface area-to-volume rate has increased. The functions and usage of nanomaterials differ according to the size and structure of NPs (Tunca, 2015). When nanomaterials are used as a biofertilizer, plants are provided with nutrients slowly, small amounts are sufficient (in contrast to chemical fertilizers), and the environmental risks caused by chemical fertilizers are minimized (Cinisli et al., 2019; Usman et al., 2020). The chemical pesticides and fertilizers adversely affect ecosystems and human health, particularly when large doses are used to increase plant yield. Therefore, it has become desirable to replace conventional pesticides and fertilizers with nanopesticides and nanofertilizers, to reduce the use of chemical fertilizers, increase plant yield, and support agricultural development (Bratovcic et al., 2021). Thus, these nanopesticides and nanofertilizers are now receiving increasing research attention (Kah, 2015). Nanofertilizers vary in size (30–40 nm), pass through the stomata, bind to different ions, and release nutrients (Bal, 2019; Cinisli et al., 2019). In general, nanofertilizers affect plant growth and metabolism by improving soil quality and plant growth performance, increasing growth hormone production and enhancing resistance to biotic and abiotic stresses (Cinisli et al., 2019; Saad et al., 2021; El-Ashry et al., 2022; Khairy et al., 2022). Nanopesticides can be produced using physical, chemical, or biological methods. Nanopesticides and nanoformulations, including those incorporating silver (Ag), Cu, silica (SiO_2_), and zinc oxide (ZnO), exhibit an improved range of pesticide efficacy compared with conventional pesticides; thus, nanopesticides positively influence the control of plant pests and diseases (Chhipa, 2017). Chitosan-metal oxide NPs have been used to ensure that the fertilizers applied to plants are taken up more effectively. The application of chitosan increases the enzyme activity of nitrate reductase, glutamine synthetase, and protease during N metabolism, thereby affecting plant growth and development (Bal, 2019). In peanut and corn plants, ZnO NPs increase the germination percentage and improve seedling development (Prasad et al., 2012; Singh et al., 2017). In addition, treatment with 2000 mg L^–1^ of 60-nm aluminum (Al) NPs for 5 days reduced the root length of corn seedlings and did not exert any adverse effects on *Lolium perenne*, *Raphanus sativus*, *Cucumis sativus*, *Brassica napus*, and *Lactuca sativa* (Yang and Watts, 2005).

Application of 2000 mg L^–1^ of Zn NPs considerably hindered root development in maize and stopped the root growth of *Brassica oleracea*, *C. sativus*, *Daucus carota*, and *Glycine max* (Lin and Zhing, 2007). Few experiments have addressed the influence of NPs on seed germination and seedling growth. However, the application of NPs on seeds generally increases seed germination, seedling development, seedling viability, and emergence rate (Abbasi Khalaki et al., 2021). Seed germination, root and shoot length, and fresh weight (FW) and dry weight (DW) values of *Agropyron elongatum* were positively affected by SiO_2_ NPs application (Azimi et al., 2014). Ag NPs have been shown to increase the germination level, length of the roots and shoots, FW and DW, average germination time, and vitality indices in *Thymus kotschyanus* (Abbasi Khalaki et al., 2016). Similarly, Ag NPs have been found to increase the germination rate in *Pennisetum glaucum* (Parveen and Rao, 2015) and *Festuca ovina* (Abbasi Khalaki et al., 2019a). However, Ag NPs can reportedly adversely affect the germination of *Brassica nigra* (Amooaghaie et al., 2015) as well as the shoot length of *Medicago sativa*, the root length and shoot DW of *Ocimum basilicum*, and the shoot and root length of *Hordeum vulgare*, *Linum usitatissimum*, and *L. perenne* (El-Temsah and Joner, 2010; Ramezani et al., 2014; Yosefzaei et al., 2016).

In *Onobrychis sativa*, SiO_2_ NPs increased shoot length, whereas titanium dioxide (TiO_2_) NPs increased germination time and percentage (Moameri et al., 2018a). Iron oxide (Fe_2_O_3_) NPs increased the germination of *L. perenne* (Wang H. et al., 2011). In addition, FeO NPs reduced the mycorrhizal biomass and the shoot and root length of *Trifolium repens* (Feng et al., 2013), *Satureja hortensis* (Peyvandi et al., 2011a), *H. vulgare*, and *L. perenne* (El-Temsah and Joner, 2010). Studies have shown that TiO_2_ NPs can positively affect the germination of *Foeniculum vulgare*, and *Petroselinum crispum* (Dehkourdi and Mosavi, 2013; Feizi et al., 2013). Ag NPs increased the shoot length and chlorophyll content of *Brassica juncea* and *Sorghum bicolor* (Namasivayam and Chitrakala, 2011; Sharma et al., 2012b). The root development of *T. kotschyanus* and *Alopecurus textilis* was positively affected by SiO_2_ NPs application (Abbasi Khalaki et al., 2019a,b). Similarly, SiO_2_ application to *M. sativa* increased plant height, tiller count, yield, FW, and DW, chlorophyll content, and carotenoid levels (Ma and Yamaji, 2006; Zmeeva et al., 2017). Govorov and Carmeli (2007) reported that SiO_2_ NPs increase leaf FW and DW as well as chlorophyll content in *O. basilicum*, and also negatively affect shoot and root growth in *S. bicolor*, *Stipa hohenackeriana*, and *Secale montanum* (Lee et al., 2012; Moameri et al., 2018b; Moameri and Abbasi Khalaki, 2019). ZnO NPs increase biomass, root and shoot length, and chlorophyll content in many plant species (Peyvandi et al., 2011b; Raliya and Tarafdar, 2013; Najafi Disfani et al., 2017; García-López et al., 2018; Yuan et al., 2018). In addition, TiO_2_ NPs increased the essential oil content and yield of medicinal plants (Ahmad et al., 2018; Fazeli-Nasab et al., 2018). The application of copper oxide (CuO) NPs adversely affects the morphology, physiology, and biochemistry of *H. vulgare*, *L. perenne*, *M. sativa*, and *Triticum aestivum* (Lee et al., 2008; Atha et al., 2012; Ramezani et al., 2014; Shaw et al., 2014; Hong et al., 2016).

### Application of NPs Under Salinity Conditions

NPs application is important to mitigate the abiotic stress effects of salinity on plants. At the germination stage, the use of Ag NPs in *Lathyrus sativus* under salt stress improves germination percentage, shoot and root length, and seedling FW and DW; thus, this enhanced osmotic regulation and reduced the negative effects associated with salinity (Hojjat, 2019). Noman et al. (2020) found that applying Cu NPs to the soil reduced oxidative stress in wheat and significantly increased plant development and yield. The use of NPs in wheat not only enhances plant development but also improves germination performance under salt-stress conditions (Shi et al., 2016). Preapplication of Ag NPs to wheat seeds alters antioxidant enzyme activities, reduces oxidative damage, and elevates salt-stress tolerance in such plants (Kashyap et al., 2015). In addition, ZnO NPs are known to increase the DW of sunflowers under salt stress (Torabian et al., 2016). CeO NPs (100 and 200 mg kg^–1^) was found to enhance the physiological responses of *B. napus* under salt stress (100 mM NaCl). CeO NPs are also known to boost plant biomass in salt-stressed canola (Rossi et al., 2016). The application of Ag NPs to basil seeds under salt-stress conditions increases seed germination (Darvishzadeh et al., 2015; Hojjat and Kamyab, 2017). Ag NPs applied to *S. hortensis* increase plant resistance to salt stress while reducing salt-stress–induced effects on germination percentage and plant shoot length (Nejatzadeh, 2021). Furthermore, the use of Ag NPs in salt-stressed cumin plants substantially improves plant salt resistance (Ekhtiyari and Moraghebi, 2012). Finally, Askary et al. (2017) reported that Fe_3_O_4_ NPs protects mint plants from oxidative stress caused by increased NaCl content.

### Application of NPs Under Drought Conditions

Drought is considered a major abiotic stress that can drastically limit crop production (Al-Ashkar et al., 2021; Roy et al., 2021). NPs application is an efficient method for alleviating the impact of drought on plants by increasing antioxidant enzyme activity, improving phytohormone levels, and affecting physiological properties. The use of analcite NPs in soil under hot, dry conditions has been shown to promote germination and plant growth in wheat (Hossain et al., 2021). In addition, the use of ZnO NPs in soybean seeds under arid conditions increases the germination percentage of the seeds (Sedghi et al., 2013). Under drought stress, the use of Cu and Zn NPs in wheat plants increases their antioxidant enzyme activity and relative moisture content, decreases thiobarbituric acid levels, affects reagent precipitation, stabilizes photosynthetic pigment levels in leaves, and reduces the effects of stress (Taran et al., 2017; Semida et al., 2021). In response to drought stress, SiO_2_ NPs application can increase shoot length and relative water content (RWC) in barley, while reducing superoxide radical formation and membrane damage (Turgeon, 2010).

Jaberzadeh et al. (2013) have reported that foliar usage of TiO_2_ NPs in wheat is effective to overcome the yield reduction caused by drought stress. Furthermore, the application of Cu NPs to maize increased leaf water content, plant biomass, and anthocyanin, chlorophyll, and carotenoid contents under arid conditions (van Nguyen et al., 2022). Ashkavand et al. (2015) reported that SiO_2_ NPs applied to hawthorn grown under drought stress reduced photosynthesis and stomatal conductivity. However, silicon (Si) NPs have been reported to ameliorate the effects of drought stress in bananas (Mahmoud et al., 2020). Under moderate drought conditions, foliar application of Si NPs to coriander resulted in optimum antioxidant capacity and essential oil yield (Afshari et al., 2021). Shallan et al. (2016) have reported that foliar application of SiO_2_ and TiO_2_ NPs can reduce the negative effects of drought stress on cotton plants under arid conditions. In chickpea plants, the application of Si NPs to the soil reduces the negative effects of drought by increasing the relative moisture content in the plants (Gunes et al., 2007). Si- and selenium (Se)-NPs can reportedly help in enhancing growth, improving ion selectivity in roots, and increasing the yield of rice under saline conditions (Badawy et al., 2021). Although drought stress increases the adverse effects of Cd in wheat, the application of ZnO NPs can reduce both Cd and drought stress (Khan et al., 2019).

### Application of NPs Under Heavy Metal Stress Conditions

Under HMs stress conditions, soil or foliar applications of NPs can eliminate the adverse effects of stress, improve plant development and photosynthesis, and reduce oxidative stress-induced toxicity. Therefore, the application of NPs contributes to in the remediation of HMs-contaminated environments. Under HMs stress conditions, the application of NPs to plants reduces the concentration of HMs in the soil, regulates the expression of HMs transfer genes in plants, increases the activity of plant antioxidant systems, improves physiological functions, and stimulates the production of protective substances such as root secretions, phytochelatin, and organic acids (Rui, 2021). The application of Si NPs on maize plants under arsenic (As) stress reduced the total chlorophyll, carotenoid content, and total protein content; in addition to mitigating the adverse effects of As stress on maximum quantum efficiency, photochemical quenching, and non-photochemical quenching of FS II (Tripathi et al., 2016). Soil application of TiO_2_ NPs can effectively limit Cd toxicity by enhancing the physiological parameters and photosynthetic rate in soybean plants; therefore, TiO_2_ NPs are vital to mitigate the effects of HMs-induced oxidative stress (Singh and Lee, 2016). When treated with SiO_2_ NPs, the activities of enzymes, such as ascorbate peroxidase (APX) and superoxide dismutase (SOD), increased; whereas the effects of oxidative stress were reduced in pea seedlings under Cr stress (Tripathi et al., 2015b). Furthermore, de Sousa et al. (2019) revealed that Si NPs can reduce Al toxicity by activating the antioxidant defense mechanism in maize plants. Konate et al. (2017) found that Fe_3_O_4_ NPs protected wheat against Cd-induced oxidative stress. Foliar applications of Se NPs to Chinese cabbage under Cd stress increased the biomass, plant height, leaf chlorophyll content, SOD levels, and plasma glutathione peroxidase (GPX) content, whereas the Cd and malondialdehyde (MDA) contents of the leaves were reduced (Zhang, 2019). Similarly, Si NPs alleviate the effect of Cd stress in rice (Wang et al., 2015). The combined use of foliar ZnO NPs and soil biochar in plants was found to be more effective against Cd stress (Rizwan et al., 2019a). Similarly, the coapplication of Fe NPs and biochar reduced the effects of Cd stress in rice (Hussain et al., 2019c). The use of FeO NPs in Cd-stressed wheat reduced the leaf electrolyte leakage ratio and Cd content in grains, while improving the antioxidant enzyme action and DW of the plants. Foliar application of Fe NPs is preferable over soil usage. Rahmatizadeh et al. (2019) also found that 20 mg L^–1^ of Fe_3_O_4_ NPs reduced Cd accumulation and improved Cd toxicity by increasing nutrient uptake in tomato plants.

### Nanofertilizers Versus Commercial Fertilizers

Agrochemicals can be released in a controlled manner, and macromolecules can be delivered selectively. By incorporating nanoscale transporters and chemicals, the efficient use of fertilizers and pesticides can be improved, resulting in a reduction in the amount used without compromising the yield of crops. In contrast, commercial fertilizers, provide fewer benefits to plants because of their larger particle size and reduced solubility. In addition, repeated chemical fertilizer application result in a toxic build-up of HMs that disrupts the ecological balance in the soil. In addition, excessive application of chemical fertilizer can contribute to soil pollution due to leaching or being not fully utilized by plants; thus, the remaining is converted into insoluble salts in the soil.

Nanoagrochemicals play an important role in enhancing nutrient use efficiency and water quality management for sustainable agriculture. However, bioaccumulation and long-term exposure of NPs to plants may have a negative impact on edible plants and food chains (Rajput et al., 2020). According to Staroń et al. (2020), NPs can be taken up and deposited in the edible tissues of crop plants. The accumulation of NPs or metal ions in their natural state can disrupt plant physiological activities; affect the integrity of cellular and sub-cellular organelle organizations; and modify the content of proteins, lipids, and nucleic acids by creating hydroxyl radicals (Cota-Ruiz et al., 2018; Rajput et al., 2020). Overall, the wide-ranging applications of NPs may generate a slew of difficulties from an ecological, ethical, health, and safety standpoint (Rajput et al., 2018).

Until now, the potential negative effects of NPs on human health have been speculative and unsubstantiated (Staroń et al., 2020). By developing various NPs as new tools for the agriculture industry, nanotechnology has grown in popularity. There is an urgent need to increase our knowledge and understanding of the specific benefits and drawbacks associated with the use of NPs. The advancement of nanotechnology has resulted in significant amounts of manufactured NPs in the agroenvironment. Although this technology has numerous advantages, researchers and experts are concerned about the unsafe disposal of NPs in large quantities (several hundred tons) each year (Rajput et al., 2020).

The existence of NPs in a various controlled objects (atmospheric air, water objects, soils, hydrobionts, algae, fungi, tissues of land plants/animals) is recommended (Rajput et al., 2020). In comparison with other sources, the fate and movement of NPs in soil have undergone very little research. Simultaneously, the soil offers fundamental nutrients to food crops, which can also operate as NPs collector sink (Rajput et al., 2020). The current review sheds light on the potential impact of NPs on the environment, health, and food security.

## Examples of NPs and the Roles They Play in Relieving Stress in Plants

### Si NPs

Si-based materials and their oxides are found abundantly in the soil. Plants naturally contain high levels of Si (1–10%) as well. Si in plants is found in the form of amorphous silica (SiO_2_⋅nH_2_O) in the cell wall, providing it with strength and solidity, in addition to contributing to polyphenols and pectins as a reactant (Bhatt and Sharma, 2018). These substances are also active during plant defense and development. Because of their application in multiple agricultural fields, it has been reported that Si-based NPs can ameliorate abiotic stresses (Jeelani et al., 2020). However, little is known about the mechanisms by which Si alleviates stresses in plants (Ma, 2004; Liang et al., 2007; Datnoff et al., 2009). Si particles and Si NPs can increase tolerance to abiotic stress, nutrient element homeostasis, stimulation of antioxidant enzymes, and improved absorption, immobilization, and partition of metal ions (Liang et al., 2007; Monica and Cremonini, 2009; Qados, 2015). SiO_2_ NPs considerably enhance germination, development, and yield in plants under stress. This can be attributed to the uptake of these NPs *via* roots leading to the development of a thin layer in the cell wall helping plants to tolerate various stresses (DeRosa et al., 2010; Siddiqui et al., 2014). SiO_2_ NPs also increase the efficiency of water translocation, increase turgor pressure, and enhance relative water inclusion in leaves and water usage effectiveness in plants (Rawson et al., 1988; Wang and Naser, 1994). Sharifi-Rad et al. (2014) found that various concentrations of SiO_2_ NPs significantly promoted maize growth and affected different developmental stages. SiO_2_ NPs can also be involved in the regulation of protein and phenolics, which are important for the growth and development of *Zea mays* (Suriyaprabha et al., 2012). In addition, they found that a relatively high level of Si accumulated in roots would boost drought tolerance in maize.

Precipitated Si NPs within plant tissues are capable of increasing the expression of essential biochemical elements, improving development, and enhancing yield factors in maize (Suriyaprabha et al., 2012). Furthermore, the improved action of the enzymatic system, the build-up of nutrients, free Pro, amino acids, and water absorption are positive effects of NPs that improve stress tolerance in crops (Wang et al., 2015; Shalaby et al., 2016; Shojaei et al., 2019). Importantly, Si NPs can also increase plant tolerance to drought stress. Ashkavand et al. (2015) observed enhanced drought tolerance as well as retention of critical biochemical and physiological attributes in hawthorn seedlings following the application of SiO_2_ NPs under different levels of drought stress. Pretreatment with SiO_2_ NPs positively influences the photosynthetic rates, stomatal conductance, and augmented xylem water potential in hawthorn seedlings under drought stress. Large dosages of SiO_2_ NPs supplied with irrigation water before drought treatments mitigate drought stress effects on growth, and biochemical and physiological parameters of *Prunus mahleb* (Tripathi et al., 2015b). Improved drought tolerance, evident by an improvement in root development and retention of the photosynthetic ratio, was also reported in two cultivars of *S. bicolor* with varying drought sensitivities after the application of Si NPs. Thus, increase in drought resistance occurred regardless of the cultivar sensitivity to drought stress (Hattori et al., 2005).

Pei et al. (2010) noted that the use of an appropriate concentration of sodium silicate (i.e., 1.0 mM) could moderately diminish the negative effects of drought stress in wheat. In the same study, there was partial promotion of shoot development and chlorophyll content when Si was supplied. This also helped retain leaf water potential and reduced membrane lipid peroxidation in stressed plants (Pei et al., 2010). Under drought stress, Si deposition in plant cells could help reduce the transpiration ratio, and enhance the photosynthesis mechanism (Ali et al., 2012; Siddiqui et al., 2014). Thus, the effects of drought stress can be mitigated by various Si/SiO_2_ NPs applications in various plant species (Zargar et al., 2010). The improved performance of such NPs can be attributed to the increased absorption and/or penetration into plant tissues; however, the exact mechanisms are not yet understood (Ashkavand et al., 2015). Shallan et al. (2016) have reported that foliar sprays of TiO_2_ NPs (50 mg L^–1^) or SiO_2_ NPs (3200 mg L^–1^) increase the drought tolerance of cotton plants. In addition, Si can help plants acclimatize to various ecological stresses (Rastogi et al., 2019). Salinity stress restrains crop yield because of Na^+^ ion toxicity in approximately 23% of planted areas worldwide (Onaga and Wydra, 2016). However, the application of Si NPs and Si fertilizer under salinity stress has positive impacts on the physiological and morphological indices of vegetative characteristics in *O. basilicum*. This is evident from the remarkable enhancement in the developmental index, chlorophyll content, and Pro concentration. This, may be because of the involvement of NPs and Si fertilizers with increasing tolerance to salinity stress in plants (Kalteh et al., 2014). The use of SiO_2_ NPs has also been shown to enhance developmental parameters, chlorophyll content, Pro accumulation, and upregulation of antioxidant enzyme activity in tomato and squash plants under salinity stress (Haghighi et al., 2012; Siddiqui et al., 2014).

The application of SiO_2_ NPs improves not only seed germination and early seedling development but also other related characteristics in lentil genotypes under salinity stress. Thus, SiO_2_ NPs boost salt toxicity protection in plants (Sabaghnia and Janmohammadi, 2014). SiO_2_ NPs can also mitigate stress by reducing Na^+^ ion concentration, resulting in improved crop development, production, and survival under salinity stress (Savvas et al., 2009). The application of SiO_2_ NPs also increases FW in maize in response to salinity stress (Gao et al., 2006). Si NPs can improve plant development by reducing osmotic potential and Na^+^ toxicity associated with high salt stress (Raven, 1983). It has been reported that SiO_2_ NPs form a layer in the root cell wall that enables plants to tolerate several stresses (e.g., salinity) (DeRosa et al., 2010; Abdel Latef et al., 2018).

Wang et al. (2010) and Wang X. et al. (2011) and others have documented the capability of Si and SiO_2_ NPs in reducing the harmful effects of salt on plant development. Because of their small size, uptaking SiO_2_ NPs can be performed more efficiently than uptaking micro-SiO_2_, -Na_2_SiO_3_, or -H_4_SiO_4_ when added to maize roots and seeds (Suriyaprabha et al., 2012). The particles were subsequently used by plants to improve growth by affecting xylem humidity, water translocation, and increasing turgor pressure; which in turn, improves the RWC and water use efficiency (WUE). The enhancement of plant germination and developmental traits by SiO_2_ NPs may be associated with an enhanced K/Na ratio, which reduces Na^+^ uptake (Alsaeedi et al., 2018), and increases the expression of antioxidant enzymes (Torabian et al., 2016; Farhangi-Abriz and Torabian, 2018). According to Almutairi (2016a), it has been found strong interactions between the enhancement of seed germination and growth in tomato-stressed plants with high salt and the increased expression of salt tolerance genes when Si NPs are applied. In contrast to no treatment of Si NPs, *Capsicum annuum* plants showed increased growth when irrigated with saline water upon the application of Si treatments (Tantawy et al., 2015).

Several studies have demonstrated that nano-Si is effective in detoxifying HMs or reducing their toxic effects while promoting plant development under HMs stress (Shen et al., 2014; Keller et al., 2015). For instance, the toxicity of Cr in pea seedlings was alleviated by supplementing the growth media with Si NPs, which reduced oxidative stress by decreasing the precipitation of Cr and increasing antioxidant mechanisms (Tripathi et al., 2015a,b). In addition, Cui et al. (2020) have reported that SiO_2_ NPs application can reduce oxidative stress in As-exposed rice cell lines. Similarly, the foliar application of 2.5 mM nano-Si can markedly increase tolerance to Cd stress in rice through the regulation of Cd precipitation (Wang et al., 2015). Si NPs have also been shown to alleviate toxicity caused by Pb, Cu, Zn, and Cd HMs, and their use may be more effective at reducing HMs accumulation compared with traditional strategies (Wang et al., 2016). Liu and Lal (2015) demonstrated that Si NPs are more effective than bulk Si in reducing the detrimental effects of Pb on rice development. Si NPs prevent Pb movement from the rice roots to the shoots and reduce Pb precipitation in grains, especially in high-Pb-precipitating cultivars and in soils with high levels of Pb contamination. Si NPs can also reduce and chelate active HMs ions, stimulate antioxidant systems, enhance the complexation and coprecipitation of toxic metals with Si, and produce fundamental changes in plants by controlling the expression of metal transport genes. However, these processes are dependent on plant genotypes, plant species, metal elements, developmental requirements, and the duration of stress enforced. Therefore, Si-mediated reductions in metal toxicity might be generalized with caution (Adrees et al., 2015). According to studies conducted by Tripathi et al. (2015b), Si NPs are linked with mitigating the toxicity effects of Cr in *Pisum sativum* seedlings. Cr stress induces toxicity; however, Si NPs can protect pea seedlings from Cr (VI)-induced phytotoxicity by reducing Cr precipitation, enhancing the antioxidant defense system, and alleviating oxidative stress. Tripathi et al. (2016) have also evaluated the effects of Si NPs on alleviating As toxicity in a maize cultivars and hybrids. Hydroponic trials have shown that these NPs can considerably reduce As toxicity by increasing the levels of metabolites of the ascorbate–glutathione cycle, and decreasing the levels of oxidative stress indicators, resulting in reduced As precipitation in the Si NP-treated cultivars and hybrids. It has been hypothesized that Si NPs can be more effective than bulk Si particles for balancing ROS production and ameliorating ROS-mediated damage in treated plants. It has also been reported that Si NPs are more effective than Si in protecting plants against UV-B stress. In general, Si NPs may protect plants by activating their antioxidant defense mechanism and regulating ROS-induced oxidative stress (Tripathi et al., 2017b).

### Ag NPs

Ag NPs are widely used in the agricultural sector, particularly in crop enhancement, food packaging, coating of domestic products, and pesticides. Their use in electronics, drug delivery, and biological-tagging medicine is also relatively common (Bechert et al., 1999; Davies, 2008; Korkin and Rosei, 2008; Jo et al., 2009; Ahamed et al., 2010; Kim et al., 2012). Ag is toxic when used in high concentrations; however, when reduced to a nanosize of 25–50 nm, it has unique properties compared with bulk Ag (Bhatt et al., 2020). Owing to these unique features, Ag NPs can be applied to enhance the vigor of plants and boost their overall development, productivity, and photosynthetic rate (Sharma et al., 2012a; Hatami and Ghorbanpour, 2013; Vannini et al., 2013; Shelar and Chavan, 2015). Ag NPs can also be used as antimicrobial substances to manage diseases on plants (Lamsal et al., 2011). The effect of different concentrations of chemically produced Ag NPs was investigated in *B. juncea* seedlings, specifically on the development and antioxidant status of the plants. Ag NPs were capable of improving growth and inducing the activity of specific antioxidant enzymes, which reduced ROS levels, improved overall antioxidant status, and reduced Pro and MDA levels. The growth-improving effect of Ag NPs in plants under stress is concentration-dependent; where a 50-ppm dose was ideal to improve growth (Sharma et al., 2012b). In another study on tomatoes, Ag NPs-treated seeds germinated earlier than those treated with deionized water; however, seed germination was inhibited when higher concentrations of Ag NPs were applied (Karami Mehrian et al., 2016).

Ag NPs may also play a role in the expression of stress genes. For instance, the up- and down-regulation of certain genes by Ag NPs was observed in microarray analysis: upregulated genes were mostly associated with responses to metal toxicity and oxidative stress, whereas downregulated genes were associated with responses to microbes and hormonal stimuli (Banerjee and Kole, 2016). Such responses may be associated plant defense mechanisms under adverse conditions; however, additional studies are required to elucidate the signaling cascades and genes controlled by Ag NPs and other NPs in various plant species.

The effects of Ag NPs on the hydraulic conductivity of the plant stem during drought stress have been studied; however, such NPs might also be capable of entering plant cells and tissues and impairing regular cellular activities (Tripathi et al., 2017a). Hojjat and Ganjali (2016) found that Ag NPs can alleviate the effect of drought stress effects in lentil (*Lens culinaris*). MahdiNezhad et al. (2018) reported that Ag NPs can reduce the levels of antioxidant enzymes in plants under drought stress; thus, this reduction can be attributed to the reduced antioxidant metabolism. NPs may be directly involved in the elimination of ROS, which reduces the levels of antioxidant enzymes. Seghatoleslami et al. (2015) reported the effects of Ag NPs on the yields and WUE of drought-stressed *Carum copticum* using a magnetic field.

Ag NPs application is useful to reduce the effect of salinity stress–induced toxicity. This has been demonstrated in studies on the germination of tomato, fennel, and cumin plants treated with Ag NPs; thus, enhancing germination, improving developmental performance, and mitigating the negative effects of salt stress (Ekhtiyari and Moraghebi, 2011; Ekhtiyari et al., 2011; Almutairi, 2016b). Positive effects of different concentrations of Ag NPs suspension have been reported on the germination and development of *Solanum lycopersicum* under salinity stress (Delfani et al., 2014). In the same study, *AREB*, *P5CS*, *MAPK2*, and *CRK1* were induced and *TAS14*, *ZFHD1*, and *DDF2* were repressed when salt-stressed plants were exposed to Ag NPs. A comparative study of the toxicity revealed that Ag NPs or AgNO_3_ had negative effects on *C. sativus* seedlings grown at higher concentrations; however, Ag NPs were less toxic than AgNO_3_ and had the potential to improve *C. sativus* yield (Cañas et al., 2008). The role of Ag NPs in relieving salt stress in wheat and *B. juncea* has been assessed, and Ag NPs were found to efficiently alleviate the effects of salinity stress (Sharma et al., 2012b; Mohamed et al., 2017; Abou-Zeid and Ismail, 2018). Ag NPs at 50 and 75 mg L^–1^ concentrations can protect plants from heat stress and improve their development (Iqbal et al., 2019).

### TiO_2_ NPs

TiO_2_ is a typical oxide of titanium. As a metal, titanium is abundant in the Earth’s crust as well as found in plant and animal tissues. TiO_2_ and nano-TiO_2_ serve as UV blockers in sunscreens because they diminish the adverse effects of UV radiation. In addition, TiO_2_ NPs have photocatalytic sterilizing properties and can undergo redox reactions when subjected to light, resulting in the formation of superoxide anion radicals and hydroxide (Hong et al., 2005). Photosterilization by TiO_2_ NPs can promote photosynthesis and improve plant growth. The potential effects of TiO_2_ NPs on the photochemical responses of chloroplasts in spinach (*Spinacia oleracea*) were evaluated (Hong et al., 2005). TiO_2_ NPs treatment was found to improve the activities of SOD, catalase (CAT), and peroxidase (POD), decrease the accumulation of reactive oxygen free radicals and MDA levels, and maintain the stability of the membrane structure of chloroplast under the light. TiO_2_ NPs also play a role in plant biochemical processes, morphophysiological characteristics, and reactions to various stresses (Mishra et al., 2014). In *S. oleracea*, TiO_2_ NPs can increase antioxidant stress tolerance through decreasing superoxide radical precipitation, reducing stress indicator (H_2_O_2_ and MDA) levels, and stimulating antioxidant enzyme activities within the plants during the photochemical interactions in chloroplasts (Lei et al., 2008).

In spinach plants, nano-anatase TiO_2_ treatment markedly increased photosynthesis, electron transmission, photoreduction activity of photosystem II, oxygen evolution, and photophosphorylation of chloroplasts under visible and UV light illumination (Lei et al., 2007, 2008). In addition, the effects of TiO_2_ NPs on plant growth have been associated with enhanced photosynthetic rate and nitrogen metabolism (Yang et al., 2006). The photocatalytic degradation of pesticides by TiO_2_ has been demonstrated as a possible water remediation process (Lee et al., 2003). Moreover, TiO_2_ NPs increase plant water uptake and nitrogen use and stimulate antioxidant activity in canola (Mahmoodzadeh et al., 2013) and wheat (Jaberzadeh et al., 2013).

Several studies have confirmed the TiO_2_ NPs-mediated improvement of plant development. For instance, Changmei et al. (2002) found that TiO_2_ and SiO_2_ NPs positively affect seed germination and growth of *G. max* (Changmei et al., 2002). In addition, onion seedlings treated with TiO_2_ NPs increased the enzymatic activity of SOD, amylase, CAT, and POD (Laware and Raskar, 2014). Mohammadi et al. (2016) explored the potential effects of different concentrations of TiO_2_ NPs against drought stress in *Dracocephalum moldavica*. Foliar application of these NPs at higher concentrations (40 ppm) can reportedly alleviate the detrimental effects of drought stress by adjusting the level of antioxidant enzymes and oxidative stress indicators. TiO_2_ NPs have been reported to increase Rubisco activase activity, chlorophyll formation, and the photosynthetic ratio and plant dry mass (Gao et al., 2008). In *Vigna unguiculata*, seed yield increases with foliar application of NPs and TiO_2_. Thus, this could be attributed to the increase in photosynthetic rates (Owolade and Ogunleti, 2008). The activity of the antioxidant enzymes (POD and CAT) increases in response to TiO_2_ NPs; therefore, MDA precipitation also decreases (Ahmad et al., 2019). The ability of TiO_2_ NPs to alleviate the adverse effects of drought stress has been investigated in several studies. For instance, the foliar application of TiO_2_ NPs can promote growth and increase the yield of wheat under drought stress when TiO_2_ NPs (0.02%) has been used (Jaberzadeh et al., 2013).

TiO_2_ NPs also improved the ability of plants to capture sunlight in maize plants. Under drought stress, TiO_2_ NPs can affect the pigment formation, the transformation of light energy to the active electron, and chemical activity, thus, enhancing photosynthetic effectiveness in maize (Akbari et al., 2014). In a similar study, the effects of nano-TiO_2_ and -SiO_2_ on the biochemical components and productivity yield of drought-stressed cotton plants have also been tested (Shallan et al., 2016). In their findings, the pretreatment with nano-TiO_2_ or -SiO_2_ can improve the pigment content, antioxidant enzyme activity, and antioxidant capacity, and increase the yield of these plants. The optimum concentrations required to reduce the destructive effects of drought stress in cotton plants were 50 and 3200 ppm for nano-TiO_2_ and -SiO_2_, respectively. Foliar application of these NPs have also increased drought tolerance in cotton plants. Similar results have been obtained in drought-stressed *L. usitatissimum* treated with TiO_2_ NPs (Aghdam et al., 2016). The drought-stressed *D. moldavica* treated with TiO_2_ exhibited increased levels of Pro and considerably reduced levels of H_2_O_2_ and MDA compared with nontreated plants (Mohammadi et al., 2014). Thus, suggesting that TiO_2_ NPs can ameliorate stress-induced damage. TiO_2_ NPs significantly induced the antioxidant enzyme activity, and Pro and soluble sugar content, which in turn promoted osmotic balance in plant cells and growth recovery in plants (Abdel Latef et al., 2018). *O. basilicum* can tolerate drought stress owing to the combined effects of gibberellin and TiO_2_ (Hatami, 2017). Overall, the application of nano-TiO_2_ can alleviate stresses of HMs, light, cold, and heat.

Singh and Lee (2016) have shown that the application of TiO_2_ NPs can reduce Cd toxicity and enhance the photosynthetic rate in soybean (Singh and Lee, 2016). TiO_2_ NPs also play an important role in alleviating light stress. When subjected to light, these NPs catalyzed the redox reaction, thereby generating superoxide anion radicals and hydroxide (Khan and Siddiqui, 2018). The addition of TiO_2_ NPs reduced the impact of heat stress by controlling stomatal opening (Qi et al., 2013). TiO_2_ NPs also positively affect plant growth and development. The positive effects of NPs, including TiO_2_-NPs, include enhancement of the carboxylation of Rubisco (Gao et al., 2006), light absorption capabilities of chloroplasts (Ze et al., 2011), electron transport rates, and prevention of ROS formation (Giraldo et al., 2014). The use of nano-TiO_2_ increases the expression level of genes encoding Rubisco- and chlorophyll-binding proteins (Hasanpour et al., 2015) as well as the activity of antioxidant enzymes (Mohammadi et al., 2014); thus maintaining stable contents of chlorophyll and carotenoids, and improving tolerance to cold conditions. Furthermore, TiO_2_ NPs positively affect susceptible (ILC 533) and resistant (Sel 11439) genotypes of chickpea under cold stress. Under such stressful conditions, TiO_2_ dramatically reduced membrane damage indices, such as ion leakage index and MDA levels, resulting in reduced damage to the membrane (Mohammadi et al., 2013). TiO_2_ treatments can also hinder oxidative damage in chickpea and reduce membrane damage under cold stress; suggesting that TiO_2_ NPs can ameliorate the redox status under heat exposure (Mohammadi et al., 2014). It has been proposed that TiO_2_ NPs improves tolerance to cold stress by enhancing the mechanisms of protection and reducing the levels of injuries in chickpea plants. Future studies may confirm the effectiveness and mechanisms of TiO_2_ NPs in improving the tolerance of crops to cold stress.

### Zn NPs

In plants, Zn is a critical micronutrient that regulates metabolic processes and facilitates development (Adhikary et al., 2010; Vitti et al., 2014). Zn also plays an important role in the survival of plants under adverse conditions. Plants use Zn in small amounts; therefore, accessibility of Zn at the nano level ensures that suitable amounts are transported to the plant while avoiding Zn toxicity in the plants and soil. ZnO is an ecofriendly compound that can be used as a “green” element (Pandey et al., 2010). Given its functions in maintaining membrane integrity, retaining the potassium content of cells, and the plant–water relationship, ZnO plays a major role in stomatal regulation (Khan et al., 2004). In a study on chickpeas, Zn deficiency decreased their ability to modulate osmotic pressure under drought stress (Khan et al., 2004). Auxin production can also be affected by Zn *via* the induction of tryptophan synthesis as a precursor for the production of indole acetic acid, which helps in root development and drought tolerance in plants (Waraich et al., 2011). The uptake of Zn can be improved when it is nano-sized; thus, the functions of Zn can be achieved more efficiently when using Zn NPs. The application of Zn NPs enhances radicle development in germinated seeds, and higher Zn content in grains; thereby improving seed survival and tolerance to environmental stresses, especially in Zn-deficient regions (Cakmak et al., 1996; Degenhardt and Gimmler, 2000; Cakmak, 2008).

Several studies have described the effects of Zn-based NPs on plant development and yield. The use of ZnO NPs, at appropriate concentrations, enhanced biomass production, seed germination, and seedling development in chickpeas, in contrast to the use of bulk ZnSO_4_. ZnO NPs can elevate auxin levels, and thus, promote plant development (Pandey et al., 2010; Burman et al., 2013). In another study, the stimulating effect of zinc sulfide (ZnS) NPs on the growth of *B. juncea* has been assessed (Nayan et al., 2016). They showed that chlorophyll content, sugar content, and plant biomass, increased significantly after the application of these NPs, and that the growth-stimulating effects were probably associated with improvements in the plant antioxidant system after ZnS NPs treatment. Furthermore, lower concentrations of ZnS NPs were more effective than higher concentrations in improving plant growth. Similar results have been reported in wheat plants treated with ZnO NPs at 66 mg L^–1^ (Awasthi et al., 2017). ZnO NPs can mediate the increases in photosynthetic pigments and a concomitant reduction in lipid peroxidation in soil-grown *Coriandrum sativum* plants (Bhatt et al., 2020). Thus, the ZnO-mediated NPs increases the photosynthetic pigments which may help plants cope with stressful conditions by stabilizing ROS generation. Sedghi et al. (2013) have reported that the germination ratio in soybean was potentially augmented by ZnO NPs under water-deficient conditions. Under drought stress, the applied ZnO NPs facilitate the rapid use of seed reservoirs for seedling development and reduced the effects of such stress (Sedghi et al., 2013).

Drought tolerance was also improved by the enhancement of antioxidant enzyme activity in wheat plants *via* ZnO NPs application (Yang et al., 2018). Both Cu NPs and Zn NPs can improve wheat plant tolerance to drought stress by enhancing the action of antioxidant enzymes and stabilizing the content of photosynthetic pigments (Taran et al., 2017). Seghatoleslami and Forutani (2015) have shown that biomass and WUE have been improved by ZnO NPs in plants under water stress, whereas plants provided with full irrigation achieved strong growth and yield with bulk ZnO treatment. Dimkpa et al. (2017, 2019) have noticed that a composite of ZnO, boric oxide, and CuO NPs can alleviate drought stress in *G. max*. Under drought stress, shoot development and grain production were enhanced by 33 and 36%, respectively, using these NPs; thus, crop productivity and uptake of P and N can be enhanced by the addition of micronutrient NPs. These findings are in agreement with those reported in another study in which ZnO NPs were demonstrated to mitigate drought-induced damage to sorghum productivity, grain fortification, and nutrient acquisition (Dimkpa et al., 2019).

Yang et al. (2018) found that remodeling of root shape by ZnO and CuO NPs could alter drought tolerance in *T. aestivum* plants colonized by *Pseudomonas chlororaphis* O6 (PcO6), a beneficial bacterial species. Zn NPs enhanced the formation of lateral roots, whereas Cu NPs stimulated the propagation of elongated root hairs close to the root tip in wheat seedlings. In the same study, the use of these NPs generally increased the expression of genes related to drought tolerance. In shoots, the expression of other genes, such as those associated with metal stress, increased, and this was consistent with the increases in Cu and Zn concentrations. Thus, plants that were subjected to CuO or ZnO NPs showed cross-protection to multiple challenges, including metal, and drought stress. Despite improvements in root hair formation and production of lateral roots caused by Cu NPs and Zn NPs, respectively, the decreased root length may be the reason for the reduction in water accessibility. In *Arabidopsis* and mustard, the increased lignification because of CuO may alter the water flow and restrict cell wall expansion. Lignification in the cell wall is a plant response that is associated with drought stress and water flow impairment; thus, this may also occur by the binding of Cu ions with cell wall pectins (Nair and Chung, 2014).

When exposed to CuO NPs, anthocyanin and Pro levels increased under water deficient stress. On the addition of CuO NPs, the precipitation of ROS improved in the roots of wheat. The increased levels of ROS and abscisic acid (ABA) due to drought stress may cause transcriptional changes, resulting in subsequent stress tolerance (Dimkpa et al., 2012). Alharby et al. (2016) have investigated the metabolic response of *S. lycopersicum* to ZnO NPs under salinity stress; and they found that the NPs can reverse the adverse effects of salinity stress by regulating tolerance-related proteins/enzymes, mainly through the upregulation of SOD and GPX gene expression. These results are consistent with those of Haripriya et al. (2018), who found that a foliar spray of ZnO NPs mitigates salinity stress effects in finger millet. ZnO NPs treatment in soil-grown sorghum can also improve drought-stress tolerance through the translocation of grain N and the restoration of total N content (Dimkpa et al., 2019). In contrast, ZnO NPs at concentrations ≥ 10 mg L^–1^ resulted in oxidative stress in tomato plants cultivated in 1/2 Murashige and Skoog media (Rédei, 2008). The differences in results could be attributed to the variation in ZnO NPs, levels of NPs used, plant development media used, and possible variation in plant liability to ZnO NPs.

ZnO NPs also reduced HMs stress by decreasing the uptake of HMs by plants; thus protecting plants from HMs toxicity (Baybordi, 2005; Venkatachalam et al., 2017). The symptoms of oxidative stress caused by Cd and Pb toxicity can be improved by ZnO NPs treatment. In addition, ZnO NPs can improve the total soluble protein and photosynthetic pigment levels, while reducing lipid peroxidation in developing seedlings of *Leucaena leucocephala* (Venkatachalam et al., 2017). When a foliar spray of ZnO NPs was applied to maize leaves, Cd absorption and Cd-induced oxidative stress were reduced (Rizwan et al., 2019c). Torabian et al. (2016) reported an increase in plant growth, photosynthetic index, and chlorophyll content and a decrease in the Na content in sunflower leaves supplied with ZnO NPs. Similarly, wheat plants treated with CuO/ZnO NPs showed improved growth, which could possibly due to the lower solubility of CuO NPs (Fathi et al., 2017). Taken together, these findings indicated that the application of Zn-based NPs enhanced plant stress tolerance.

## Nanotechnology-Based Advances in Agriculture

Nanotechnology has several other possible applications and can play an important role in agriculture, forestry, energy production, food processing, environmental management as well as in ensuring water quality and utilizing waste resources (Prasad et al., 2017; Kim et al., 2018). The extensive scope of nanotechnology and its wide range of applications has led to advancements in the agricultural sector (Kim et al., 2018; Shang et al., 2019). Over the last two decades, the use of nanotechnology in agriculture has been supported by research and practical applications at the academic and industrial levels (Shang et al., 2019).

In particular, nanotechnology has been applied to increase crop production. In addition, nanotechnology has been used to produce nanofertilizers and nanoencapsulated nutrients, which are considered promising methods for achieving site-targeted and regulated delivery of nutrients to plants, thereby improving crop production and yield *via* “precision agriculture.” Nanoformulations of agrochemicals, such as nanopesticides and nanofertilizers, substantially reduce micronutrient losses of fertilizers through volatilization and leaching, enhance effective phytoavailability, feed plants gradually in a controlled manner, and eventually reduce environmental hazards caused by the excessive use of traditional fertilizers (Solanki et al., 2015; Shang et al., 2019; Zulfiqar et al., 2019). Nanofertilizers can be produced using Cu, SiO2, Zn, TiO_2_, and polymeric NPs as dendrimers acting as nanocarriers (Paramo et al., 2020).

Studies have shown that nanofertilizers can help crop productivity by improving stress tolerance as well as promoting plant germination, growth, and physiological processes. However, nanofertilizers have some drawbacks that have restricted their extensive application (Zulfiqar et al., 2019). Nanosensors have been reported as another application of nanotechnology that can improve crop quality and yield, while ensuring an output of high-quality and healthy food products. Novel nanosensors are primarily applied in crop safety for the detection and management of phytopathogens and for measuring and monitoring the uses, penetration, and residues of agrochemicals as well as environmental pollution (Ion et al., 2010; Chen et al., 2016; Prasad et al., 2017; Paramo et al., 2020). Their use has advanced the human management of soil and plant health, improved food quality, and protection, optimized packaging methods, and enhanced soil monitoring and crop conditions (Kim et al., 2018; Shang et al., 2019). Other agronomic uses of nanotechnology include the use of nanodevices in plant genetic engineering, postharvest management, and plant disease diagnostics (Ion et al., 2010; Prasad et al., 2017). Nanobiotechnology includes the use of novel methods to genetically modify and engineer crop programs that boost agricultural productivity, food safety, and processing capacity while promoting agricultural sustainability.

**FIGURE 4:**
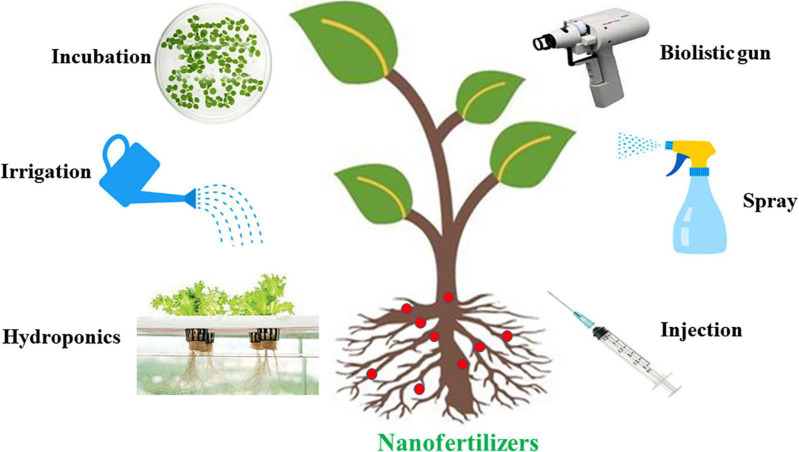
Methods used for the application of nanoparticles in the field of agriculture.

Different methods for the application of NPs in agriculture are shown in Figure 4. The application of nanomaterials enable efficient plant transformation for crop improvement (Anderson et al., 2016; Shafiee-Jood and Cai, 2016). Given their unique properties of small size, multiple binding sites and large surface area, nanomaterials are excellent nanocarriers of bioactive molecules (e.g., plasmid DNA and double-stranded RNA) (de Oliveira et al., 2014; Anderson et al., 2016; Shafiee-Jood and Cai, 2016; Kim et al., 2018; Zhao et al., 2020). Engineered NPs can also be used to increase crop safety and detect pesticide residues (Kim et al., 2018). Moreover, nanotechnology has become a common method used by engineers and designers to enhance and improve soil properties. Nano clay–polymer composites and nano-zeolites can be used in the soil to improve its moisture content, increase water-retention capacity, and slow water release during the cultivation season, and nanomagnets have been used to expel soil contaminants (Vundavalli et al., 2015; Prasad et al., 2017; Kim et al., 2018).

The application of nanofertilizers can also help reduce soil toxicity caused by an accumulation of chemical substances applied to the soil, while also acting as an alternative means of enhancing resource-use efficiency (DeRosa et al., 2010; Nair et al., 2010; Jalil and Ansari, 2019). In addition, nanosensors are now widely used in agriculture for soil analysis, water management and transmission, environmental monitoring of pollution in soils and water, and pesticide and nutrient drop (Ion et al., 2010). Various sensors based on nanodetection technology, including biosensors, optical sensors, electrochemical sensors, and other instruments, are important tools for identifying HMs at trace levels (Ion et al., 2010; Prasad et al., 2017; Singhal et al., 2022).

## Mechanisms of Action of NPs in Plants

Although NPs have a wide range of applications in agriculture, the majority of NPs are hazardous to plants when present in high concentrations. The uptake, accumulation and interference of NPs with key metabolic processes in different plant tissues may have positive or negative effects on plants, depending on their dosage, movement, characteristics, and reactivity.

### Uptake of NPs

High concentrations of NPs can penetrate plant cells and cross the plasma membrane; thus, this may interfere with key cellular activities (Mazumdar and Ahmed, 2011; Mirzajani et al., 2013). NPs can reach plant tissues through the root system or above-ground parts such as root junctions and wounds. As a carrier, NPs must pass through several physiological barriers until they are taken up by the plant and translocated. Plant cell walls, which are made up of cellulose, allow small NPs, ranging between 5 and 20 nm in size, to pass through into the plant cells (Dietz and Herth, 2011).

Some NPs have been shown to develop larger pores in the cell wall to enter the cell (Navarro et al., 2008; Kurepa et al., 2010). NPs can be transferred to other plant tissues *via* the apoplastic and symplastic pathways (Etxeberria et al., 2006; Ma et al., 2010). Wong et al. (2016) suggested a lipid exchange mechanism for NPs transport into plant cells. The size, magnitude, and zeta potentials of NPs are important to determine their delivery in plant cells.

### NPs-Plant Interaction Pathways

NPs may affect plant metabolism by delivering micronutrients (Liu and Lal, 2015), gene regulation (Nair and Chung, 2014), and interfering with several oxidative processes in plants (Hossain et al., 2015). Excessive contents of NPs can generate ROS; thus, interfering with the oxidative mechanism; while other pathways have yet to be deciphered. The NPs can disrupt the electron transport chain in mitochondria and chloroplast, causing an oxidative burst and an increase in ROS levels (Pakrashi et al., 2014; Cvjetko et al., 2017). The rate of carbon fixation is reduced in response to stressful conditions; thus, this increases photoinhibition, potentially leading to the overproduction of superoxide anion radicals and H_2_O_2_ in the photosystem (Foyer and Noctor, 2005). When ROS is generated as a result of NPs, all biological components are affected causing protein changes, lipid peroxidation, and DNA damage (Van Breusegem and Dat, 2006).

Several studies have found an increase in lipid peroxidation and DNA damage in plants while interacting with NPs (Atha et al., 2012; Saha and Dutta Gupta, 2017). The increase in ROS levels can cause apoptosis or necrosis, resulting in plant cell death (Faisal et al., 2013). Despite its destructive nature, ROS play a role in biological activities, including stress tolerance (Sharma et al., 2012a). The balance between ROS generation and scavenging determines whether ROS has a destructive or signaling function. The cells have developed a robust antioxidant mechanism to precisely control the quantity of ROS. Enzymatic (SOD, CAT, and guaiacol peroxidase) and non-enzymatic (ascorbate, glutathione, carotenoids, tocopherols, and phenolics) antioxidants are attributed to defense mechanisms in plants (Sharma et al., 2012b). Several studies have demonstrated that plants exposed to NPs produce more antioxidant molecules (Jiang et al., 2014; Costa and Sharma, 2016). Plant stress response signaling can also be influenced by phytohormones (Mengiste et al., 2010; O’Brien and Benková, 2013; Sham et al., 2019).

Plant hormones are endogenous molecules involved in the regulation of plant development and stress tolerance (Sham et al., 2017). In response to abiotic stresses, different hormonal pathways can be activated or suppressed (Kwak et al., 2006; O’Brien and Benková, 2013). In red pepper (*Capsicum annuum*), cytokinin levels increased in response to AgNPs stress; while in cotton (*Gossypium* sp.), a decrease in the levels of auxins and ABA in response to CuO NPs was detected. This suggests that NPs affect plant hormonal balance and plant metabolism.

Several studies have demonstrated that NPs can also affect the content and activity of photosynthetic pigments in plants (Perreault et al., 2014; Tripathi et al., 2017c). High concentrations of NPs have a negative impact on photosynthesis, resulting in growth retardation or death in plants (Tripathi et al., 2017c).

## Future Prospects on NPs for Enhancing Crop Tolerance to Abiotic Stress

Nanobiotechnology has the potential to improve stress tolerance, stress sensing/detection, targeted delivery and controlled release of agrochemicals, transgenic events, and seed nanopriming in plants (Wu and Li, 2022). Such nanomaterials free of HMs and high dispersibility can be developed for agricultural use. Future research on evaluating the biological effects of nanozymes i.e., Mn_3_O_4_ NPs in plants under stress conditions should be on top of our priorities. Mechanisms underlying nanopriming-induced seed germination, breaking seed dormancy, and their interactions with seeds have to be investigated. Understanding how NPs improve plant stress tolerance will enable researchers to design tailor-made nanomaterials targeting agricultural challenges. In addition, nanomaterials have no doubt a bright future ahead, especially when it comes to their functionality in plants. For example, Santana et al. (2020) have developed a targeted delivery approach using nanomaterials to convert chloroplasts into “chloroplast factory” for better plant photosynthesis under low light conditions. The use of nanomaterials for CRISPR-Cas genome editing in cargo delivery (Demirer et al., 2021) will increase the efficiency of genetic engineering to enhance plant stress tolerance. Developing policies and regulations could help manage biosafety hazards associated with the use of nanomaterials in agriculture. We believe that nanomaterials will play a crucial role in the future of agriculture.

## Conclusion

To achieve sustainable agriculture, the research community must identify appropriate ecofriendly solutions that address abiotic-stress–induced loss of crop yield (Figure 3). Nanotechnology is an innovative and effective means of promoting crop yield and quality, enhancing the farming sector, and managing global food demand. The potential role played by several NPs in alleviating abiotic stress-induced damage and improving plant development and crop yield is under intense investigation. NPs, such as TiO_2_, SiO_2_, and Ag NPs, can reduce the negative effects of abiotic stress by activating plant defense mechanisms *via* the induction of ROS production and phytotoxicity. NPs, given their small size, can also easily penetrate plant tissues, after which they positively influence plant morphological, physiological, and biochemical processes, promote plant development, and improve crop productivity in plants under various abiotic stresses. Moreover, NPs have a large surface area that improves the absorption and delivery of various targeted nutrients. Nevertheless, the applications of NPs in crop improvement and sustainable agriculture are still at an early stage of development, and the current research in the field is insufficient and, to some extent, inconsistent (Rajput et al., 2021). Therefore, additional investigations must explore the following issues, which will help limit the undesirable effects of NPs on ecosystems and crops: (a) the reaction of NPs with plants and metabolic process at the molecular and cellular levels, and optimization of NPs size and level before practical application in the field; (b) the effects of NPs and their possible toxicities in different plant species; (c) the impact of NPs on gene regulation and expression in plants under various abiotic stresses; (d) the behavior and fate of NPs in plants and the environment; (e) the effects of soil properties and different plant species on the efficiency of NPs; (f) the classification of NPs as stress initiators or stress in activators; and (g) the combined effects of NPs with other active ingredients and biotic stresses in plants.

## Author Contributions

All authors listed have made a substantial, direct, and intellectual contribution to the work, and approved it for publication.

## Conflict of Interest

The authors declare that the research was conducted in the absence of any commercial or financial relationships that could be construed as a potential conflict of interest.

## Publisher’s Note

All claims expressed in this article are solely those of the authors and do not necessarily represent those of their affiliated organizations, or those of the publisher, the editors and the reviewers. Any product that may be evaluated in this article, or claim that may be made by its manufacturer, is not guaranteed or endorsed by the publisher.
